# Post-Pandemic Epidemiology of Respiratory Infections among Pediatric Inpatients in a Tertiary Hospital in Shanghai, China

**DOI:** 10.3390/children11091127

**Published:** 2024-09-17

**Authors:** Siyuan Lan, Changjuan Gu, Shuanglong Lu, Ning Zhou, Xiaohong Qiao

**Affiliations:** Department of Pediatrics, Tongji Hospital, School of Medicine, Tongji University, Shanghai 200065, China; 2411226@tongji.edu.cn (S.L.); 2000655@tongji.edu.cn (C.G.); 1400456@tongji.edu.cn (S.L.); 024406@tongji.edu.cn (N.Z.)

**Keywords:** children, COVID-19, epidemiology, pathogen, respiratory infection

## Abstract

Background: After the removal of the three-year epidemic control restrictions, Chinese children were confronted with heightened risks of respiratory infections. We aimed to investigate the post-pandemic (2023) epidemiology of respiratory infections among pediatric inpatients in a tertiary hospital in Shanghai, China, and compare it with the pre-pandemic (2019) levels. Methods: A total of 2644 pediatric inpatients were enrolled based on discharge time and divided into group 2019 (*n* = 1442) and group 2023 (*n* = 1202). Information on the demographic characteristics, diagnoses, and pathogen test results (Mycoplasma pneumoniae, MP; Chlamydia pneumoniae, CP; Legionella pneumophila, LP; Influenza A, IFA; Influenza B, IFB; Parainfluenza virus, PIV; respiratory syncytial virus, RSV; Coxsackie virus, COX; Adenovirus, ADV; Epstein–Barr virus, EBV) was collected and analyzed. Results: Significant increases were found in the overall test positivity rates (64.6% vs. 46.7%), mixed infection rates (17.4% vs. 9%), and proportion of severe cases (25.5% vs. 3.7%) after the pandemic than those before it. Compared with 2019, the incidences of MP, IFA, LP, RSV, and ADV remarkably increased, while those of IFB and COX decreased, with no obvious differences noted for CP, PIV, and EBV in 2023. A significantly higher MP-positive detection rate was noticed in children aged 1–6 years in 2023 than in 2019. The incidence of RSV infection began to rise in August 2023, earlier than the conventional epidemic season. Conclusions: Compared with the pre-pandemic levels, the overall test positivity rates of atypical pathogens and viruses among pediatric inpatients significantly increased, and alterations in the disease spectrum, epidemic season, and age of prevalence were observed after the COVID-19 pandemic.

## 1. Introduction

Acute respiratory tract infections (ARIs) caused by bacteria, viruses, and atypical pathogens are the primary reasons for children’s medical consultation and hospitalization worldwide [1,2]. The implementation of non-pharmaceutical interventions (NPIs) during the 2019 novel coronavirus (COVID-19) pandemic, including quarantine measures, social distance norms, and mask mandates, effectively blocked the transmission of respiratory pathogens and led to historically low levels of ARI prevalence [2,3,4,5,6]. 

At the end of 2022, the rapid spread of the highly contagious Omicron variant placed heavy burdens on social and economic development [7]. In consideration of high vaccination rates for COVID-19 and the low virulence of the Omicron variant in China, the three-year epidemic control restrictions were removed [7,8], and multiple kinds of respiratory pathogens swooped in thereafter. The resurgence of seasonal viruses and a shifted infection spectrum had already arrested extensive attention in other post-pandemic countries [2,4,9,10], which might be attributed to the “immunity debt”, a phenomenon characterized by diminished herd immunity due to prolonged low exposure to viruses and bacteria during epidemics [9,10,11]. Immunologically immature children were confronted with heightened risks.

It still remains uncertain how long this situation will persist and when it will revert to the pre-pandemic levels. And most of the previous studies only concentrated on one or several common viral infections, while neglecting the atypical pathogens and less prevalent viruses. Having implemented NPIs for the longest duration, it is imperative for China to comprehensively monitor the epidemiology of ARIs in children. 

The objectives of this study were to investigate the post-pandemic (2023) epidemiology of respiratory infections caused by atypical pathogens and viruses in pediatric inpatients in a tertiary hospital in Shanghai, China, and compare it with the pre-pandemic (2019) levels.

## 2. Materials and Methods

### 2.1. Study Design

This retrospective study was conducted in the Department of Pediatrics at Shanghai Tongji Hospital, a tertiary general hospital in Shanghai, China. The inclusion criteria were patients aged over 1 month and under 14 years old, discharged before (from 1 January to 31 December in 2019) and after (from 1 January to 31 December in 2023) the pandemic, and diagnosed with ARI (upper respiratory infection, laryngitis, bronchitis, bronchiolitis, and pneumonia). Children with immune deficiency, hematological disorders, congenital malformations, genetic defects, or incomplete medical records were excluded. Information including age, gender, diagnoses, length of stay, and pathogen test results of these inpatients was collected through electronic medical records. The severity of disease was assessed based on the Chinese guideline for the diagnosis and treatment of community-acquired pneumonia in children [12]. 

In our hospital, for children in which COVID-19 was positively detected, mild patients were treated in the outpatient and emergency departments, while severe patients were transferred to designated COVID-19 specialized hospitals with isolation wards for treatments. Therefore, COVID-19-positive cases were excluded from our study.

### 2.2. Sample Collection and Laboratory Test

Patients with ARIs were approached for the collection of throat swabs and serum samples for pathogen testing within 6 h of admission before treatments. Pharyngeal swab specimens were sent for the detection of influenza antigens by the colloidal gold method (Innovita, Beijing, China). Serum samples were sent to test for the IgM antibodies of Mycoplasma pneumonia (MP), Chlamydia pneumonia (CP), Legionella pneumophila (LP), Influenza A (IFA), Influenza B (IFB), Parainfluenza virus (PIV), respiratory syncytial virus (RSV), Coxsackie virus (COX), and Adenovirus (ADV) using indirect immunofluorescence assays (Autobio, Shanghai, China), and Epstein–Barr virus (EBV) was tested for using a chemiluminescence assay (Snibe, Guangzhou China). The level of MP antibody titer was also measured, and an MP titer above 1:160 was considered a positive result. A positive nasopharyngeal swab or serology can both diagnose influenza. The detection of MP DNA, A2063G, and A2064G mutations was conducted on children suspected of macrolide-resistant MP (MRMP) infection and capable of retaining sputum specimens, performed by real-time polymerase chain reaction (Kingmed, Guangzhou, China).

### 2.3. Statistical Analysis

The categorical variables were presented as numbers (percentages), and the continues variables were given as the median (interquartile range). Categorical variables were compared using the Chi-square test or Fisher’s exact test with limited data availability. Continuous variables were compared using the Mann–Whitney U test. A *p* value < 0.05 was considered statistically significant. All statistical analyses were performed using the SPSS statistical software package version 22.0 (SPSS Inc., Chicago, IL, USA).

## 3. Results

The demographic characteristics and pathogens of hospitalized children before (2019) and after (2023) the pandemic are presented in Table 1. The eligible subjects were divided into group 2019 (*n* = 1442) and group 2023 (*n* = 1202) based on discharge time, with median ages of 4.1 years and 6.4 years, respectively. Significant differences in the age structure were observed between these two groups. Preschool children accounted for a large proportion (38.9%) in 2019, while the 2023 group was mainly composed of school-age and adolescent children (55.2%). The hospitalization time in 2023 was significantly shorter than that in 2019, despite an obviously growing number of severe and co-infection cases. Compared with group 2019, significantly higher rates of MP, IFA, LP, RSV, and ADV infections were found in group 2023, while lower rates of IFB and COX infection were detected. Only two cases of MRMP were observed in 2019. There were no differences in the incidences of CP, PIV, and EBV between the two groups.

**Figure 1:**
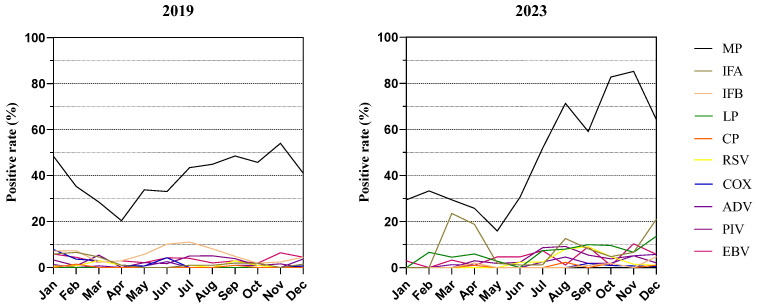
The overall changes in the prevalence of respiratory pathogens among pediatric inpatients before (2019) and after (2023) the pandemic. Mycoplasma pneumoniae, MP; Influenza A, IFA; Influenza B, IFB; Chlamydia pneumoniae, CP; Legionella pneumophila, LP; respiratory syncytial virus, RSV; Coxsackie virus, COX; Adenovirus, ADV; Parainfluenza virus, PIV; Epstein–Barr virus, EBV.

**Figure 2:**
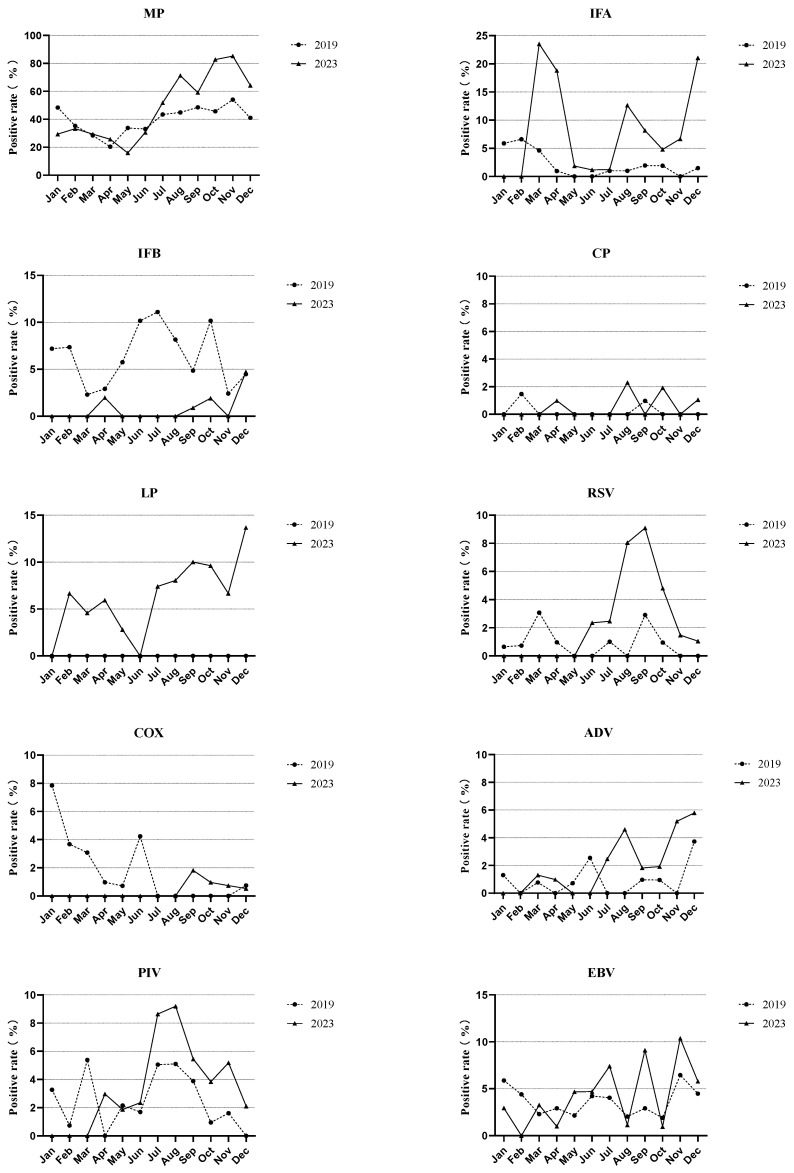
Changes in the prevalence of each respiratory pathogen among pediatric inpatients before (2019) and after (2023) the pandemic. Mycoplasma pneumoniae, MP; Influenza A, IFA; Influenza B, IFB; Chlamydia pneumoniae, CP; Legionella pneumophila, LP; respiratory syncytial virus, RSV; Coxsackie virus, COX; Adenovirus, ADV; Parainfluenza virus, PIV; Epstein–Barr virus, EBV.

In Figure 1 and Figure 2, the positive rate of MP stayed consistently high, mostly exceeding 20% and peaking at over 80% in the latter half of 2023. IFB was prevalent during the summer of 2019, while IFA had an outbreak in the spring of 2023. The infection rates of CP were below 3% in both groups. No positive cases of LP were examined in 2019. The vertices of the RSV, COX, and PIV curves occurred in September 2023, January 2019, and August 2023, respectively. 

**Figure 3:**
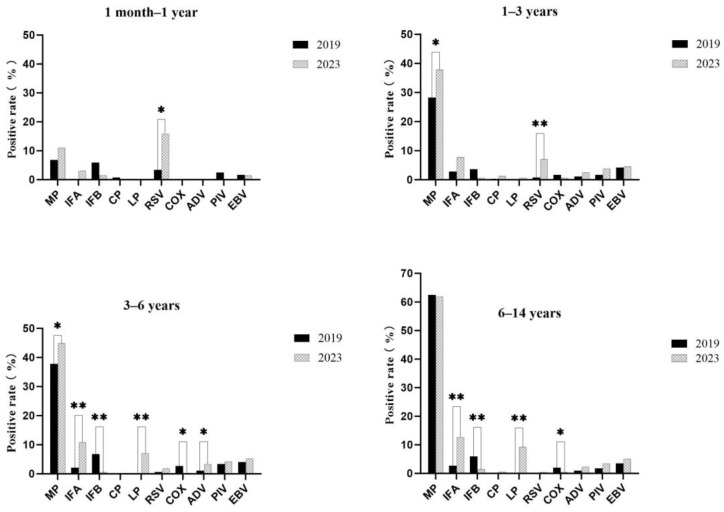
The positive detection rates of respiratory pathogens among pediatric inpatients of different age groups before and after the pandemic. Mycoplasma pneumoniae, MP; Influenza A, IFA; Influenza B, IFB; Chlamydia pneumoniae, CP; Legionella pneumophila, LP; respiratory syncytial virus, RSV; Coxsackie virus, COX; Adenovirus, ADV; Parainfluenza virus, PIV; Epstein–Barr virus, EBV. * *p* < 0.05; ** *p* < 0.001.

The positive detection rates of respiratory pathogens among pediatric inpatients of different age groups before and after the pandemic are displayed in Figure 3. In comparison with 2019, there were noticeable increases in RSV infection among children under the age of 3 as well as MP infection among children aged 1 to 6 in 2023. In pediatric patients aged 3–14, significantly higher proportions of IFA and LP infections but lower proportions of IFB and COX infections were found in 2023 compared to 2019. There were no discrepancies in the occurrences of CP, PIV, and EBV infections across all age groups. 

The demographic characteristics and pathogen test results of severely hospitalized children before and after the pandemic are described in Table 2. In 2023, the median age of severe pediatric patients and the percentage of children over 3 years old significantly increased compared to 2019, coinciding with surges in MP and MRMP cases. The pathogen detection rates of severe cases improved remarkably, leading to a reduction in hospital stay duration in 2023. More cases of LP but fewer cases of IFB were detected in critically ill children in 2023 compared to 2019.

As Table 3 shows, the median age of children infected with MRMP was 8.7 years old, of which, 61% (119/195) were identified as severe cases. Patients suffering from severe MRMP infection experienced longer hospitalization stays than the non-severe group. The most common co-infection with MRMP was EBV (8.7%).

The percentages of severe cases of each pathogen before and after the pandemic are exhibited in Table 4. MP infection was responsible for the highest number (258) of severe cases in 2023. Compared with 2019, significantly higher rates of severe cases caused by MP and EBV were noticed in 2023. No significant differences were found in the severity rates of other pathogens before and after the pandemic.

## 4. Discussion

Throughout the three-year COVID-19 pandemic, Shanghai rigorously adhered to the dynamic zero policy and implemented 7–14-day quarantines for locations where positive cases were identified [8]. The data from 2019 to 2022 in this investigation were intermittent due to multiple quarantine periods, thereby failing to mirror the actual situation of RTI in pediatric inpatients. Additionally, considering that numerous domestic and foreign studies had confirmed a significant decrease in respiratory infectious diseases after the implementation of NPI [2,3,4,5,6], we excluded the information during the epidemic period and instead emphasized the alterations before and after the pandemic.

This article focused on the post-pandemic epidemiology of respiratory infections in pediatric inpatients in mainland China, covering a wide range of pathogens including MP, CP, LP, IFA, IFB, PIV, RSV, COX, ADV, and EBV. The overall positive detection rate of respiratory pathogens among hospitalized children in Shanghai significantly escalated (64.6% vs. 46.7%) after the epidemic compared to before it, along with higher proportions of severe cases (25.5% vs. 3.7%) and mixed infections (17.4% vs. 9%). A peak of IFA infection emerged in the early stage of 2023, followed by an outbreak of MP infection that occurred in the second half of that year. The infection rates of seasonal viruses such as IFA, ADV, and RSV remarkably elevated, yet there were no substantial differences in the positive rates of CP, PIV, and EBV in 2023 compared with 2019.

### 4.1. Atypical Pathogens

The widespread outbreak of MP in China since July 2023 had garnered significant attention [13], and a similar resurgence of MP infections in children was also witnessed in the United States in autumn 2023 [14], particularly the prevalence of MRMP. MRMP was first reported in 2000 and exhibited an overall ascending trend across the world [15,16]. Heng Li et al. disclosed that MRMP had been spreading across China before 2020, but NPIs during the pandemic delayed the outbreak of MRMP until 2023 [13]. Despite regional disparities, previous data suggested that the overall proportion of MRMP in China had reached 79.5%, the highest worldwide [17]. In this study, the percentage of MRMP among hospitalized children rose from 0.34% (2/573) to 73.9% (193/261) from 2019 to 2023. Given that some of the young patients were unable to successfully retain sputum samples, the actual infection rate of MRMP ought to be even higher. 

Children infected with MRMP are predisposed to experiencing refractory MP (clinical symptoms and imaging examination results unrelieved after regular treatments for 7 days), leading to a constellation of extrapulmonary complications and bringing substantial pressure to the pediatric outpatient clinics and wards [18]. Our research indicated that the vast majority (89%) of severe cases in 2023 were attributed to MP infection, and among patients with positive detection for MRMP, 61% were identified as severe cases. Furthermore, it is notable that the median age of hospitalized patients became significantly older (6.4 years vs. 4.2 years), as MP is usually more prevalent among school-age children [17]. However, there was also an increase in MP infection incidence among children aged 1–6 years in 2023, implying that this MP outbreak had exerted an impact on children across all ages. 

The morbidity of CP stayed at relatively low levels throughout the year, with the lowest positive detection rate (0.4%) among all pathogens and no association with severe cases. A retrospective study by Fengqing Cai et al. demonstrated a reduction in the number of CP-positive patients during the pandemic [19], and our research suggested that there was no remarkable difference in the infection rate of CP between 2019 and 2023. 

LP is rarely seen in children, and the incidence of LP in Korean children was reported to be 0.02 per 100,000 individuals by the Korea Centers for Disease Control and Prevention in 2017 [20]. No positive case of LP was identified in 2019 within our study. Nevertheless, the positive rate of LP increased significantly in children over three years of age and was correlated with severe infection in 2023. In addition to the effect of the immunity debt, we also considered that the increments in the overall test positive rates of respiratory pathogens and the proportion of mixed infections in 2023 might have led to some false positive results, owing to the antigen cross-reactivity between LP and other pathogens such as *Streptococcus pneumoniae* and *Bacteroides fragilis* [21]. On the other hand, the absence of titration for LP antibodies could also undermine the credibility of the findings. Hence, multi-centered studies and more precise detection methods are requisite to evaluating the epidemic trends in LP.

### 4.2. Enveloped Viruses

Enveloped viruses are more sensitive to environmental disinfection and hand hygiene measures than non-enveloped viruses, and thus, their incidence rebounded rapidly subsequent to the mitigation of NPIs [3]. IFA is highly mutable and capable of triggering a larger-scale epidemic compared to IFB [22]. In our research, IFA was the first virus to prevail in 2023, while IFB failed to reach the pre-epidemic level. IFA and IFB accounted for 5.8% and 1.4% of severe cases in hospitalized children, respectively. It is worth noting that there is no cross-immunization between IFA and IFB [22], highlighting the significance of timely vaccination with influenza vaccines in advance to prevent the dissemination of influenza and minimize severe cases. 

RSV infection is a major contributor to wheezing and severe pneumonia in children under 3 years old [9]. Meanwhile, our investigation revealed notable increases in the incidence of RSV infection (2.5% vs. 0.8%) in 2023 compared with those in 2019. A Pierangeli et al. pointed out that the post-epidemic RSV peak was the consequence of virus mutation and immunity debt created by disrupted RSV cycling [23]. The first encounter with RSV after the pandemic in children under 3 years old was able to readily evolve into severe infections as they had not yet developed their own antibodies or received them from their mothers via the placenta [9]. Although the positive detection rate of RSV in children under 3 years old significantly rose in 2023, this group merely constituted a small proportion (9.5%) in our study. Consequently, there were not many severe cases caused by RSV. The epidemic season of RSV in this study occurred in August and September, earlier than the conventional infectious period spanning from November to February. Similarly, the increases in RSV-positive cases since April 2021 in the United States [24] and September 2022 in Germany [1] were both reported after the pandemic. This phenomenon reminds healthcare institutes to be vigilant against off-season viruses in children with RTI, although conducting additional pathogen tests may impose economic burdens on patients. 

The positive detection rates of PIV and EBV both showed no significant differences before and after the pandemic in our data. Lifeng Li et al. found that the incidence of PIV infection hit its nadir in 2020 in China, succeeded by a small rebound in 2021 and 2022, but still remaining beneath pre-epidemic levels [25]. However, the impact of COVID-19 on the positive detection of EBV in children has been scarcely discussed in the literature. Unlike other respiratory pathogens, EBV is not transmitted through aerosols but rather through close contact with saliva, so its spread was less influenced by NPIs. In this study, the increase in the severity rate of EBV in 2023 may have been associated with the number of cases co-infected with MRMP. The overall EBV positivity rate among the subjects was 4.3%, lower than the 7.3–14.3% reported in other studies [26]. This might have been the result of the exclusive identification of EBV antibodies without the determination of DNA in our study. In addition, it was hypothesized that the reactivation of EBV induced by COVID-19 may be related to long COVID [27], for which the underlying mechanism needs to be elucidated in the future. Long COVID and the immune dysfunction resulting from COVID-19 infection [28] might constitute some of the reasons for the increased incidence of respiratory infectious diseases.

### 4.3. Non-Enveloped Viruses

Numerous studies have documented that non-enveloped viruses such as ADV, rhinovirus, and enteroviruses endured throughout the pandemic and recovered shortly after the easing of NPIs [2,3,4]. In 2023, the ADV positivity rate in this study surpassed the pre-pandemic level. However, the detection rate of COX was lower than that in 2019, and no positive cases were detected until September 2023. COX is one of the enteroviruses with various genotypes, capable of causing diverse symptoms and illnesses in children [29]. One possibility is that different COX subtypes contributed to the discrepancies between 2019 and 2023, which could not be distinguished by the serological detection methods applied in this research. Currently, limited data have concentrated on the post-pandemic epidemiology of COX infection in children, and further investigations are needed to determine whether these outcomes stem from the epidemic characteristics of the virus itself or potential confounding factors. 

This article has certain limitations, as it is a single-center, retrospective study with limited detection approaches and lacks information on COVID-19-positive cases, COVID-19 vaccinations, and bacterial infections. However, it provides information on the epidemiological characteristics of a wide range of respiratory pathogens in Chinese children, including the relatively less-discussed LP, COX, and EBV, and offers valuable insights for developing strategies to prevent and control pediatric infectious diseases in the post-pandemic period.

## 5. Conclusions

Compared with the pre-epidemic period, the positive detection rates of respiratory pathogens, the proportions of mixed infections, and severe cases among hospitalized children significantly increased after the lifting of NPIs in our study. The incidences of MP, IFA, LP, RSV, and ADV increased remarkably, while those of IFB and COX decreased, with no obvious differences noted for CP, PIV, and EBV. The alterations in disease spectrum, the off-season circulation, and the expanded susceptible age range were the new features of pediatric respiratory infections. 

## Figures and Tables

**Table 1:** The demographic characteristics and pathogen test results of pediatric inpatients before (2019) and after (2023) the pandemic.

Characteristics	Total (*n* = 2644)	2019 (*n* = 1442)	2023 (*n* = 1202)	*p* Value
Male (%)	1415 (53.5)	776 (53.8)	639 (53.2)	0.738
Age (y)	5 (2.9, 7.7)	4.1 (2.4, 6.4)	6.4 (3.8, 9)	<0.001
<1 y (%)	181 (6.8)	118 (8.2)	63 (5.2)	<0.001
1 y–3 y (%)	514 (19.4)	361 (25)	153 (12.7)	<0.001
3 y–6 y (%)	883 (33.4)	561 (38.9)	322 (26.8)	<0.001
≥6 y (%)	1066 (40.3)	402 (27.9)	664 (55.2)	<0.001
Length of stay (d)	6 (5, 7)	6 (5, 8)	5 (4, 7)	<0.001
Severe cases (%)	361 (13.7)	54 (3.7)	307 (25.5)	<0.001
Mixed infections (%)	339 (12.8)	130 (9)	209 (17.4)	<0.001
Positive rate (%)	1451 (54.9)	674 (46.7)	777 (64.6)	<0.001
MP (%)	1194 (45.2)	573 (39.7)	621 (51.7)	<0.001
MRMP (%)	195 (7.4)	2 (0.1)	193 (16.1)	<0.001
IFA (%)	166 (6.3)	33 (2.3)	133 (11.1)	<0.001
IFB (%)	96 (3.6)	82 (5.7)	14 (1.2)	<0.001
CP (%)	10 (0.4)	3 (0.2)	7 (0.6)	0.118
LP (%)	86 (3.3)	0 (0)	86 (7.2)	<0.001
RSV (%)	42 (1.6)	12 (0.8)	30 (2.5)	0.001
COX (%)	34 (1.3)	29 (2)	5 (0.4)	<0.001
ADV (%)	45 (1.7)	14 (1)	31 (2.6)	0.001
PIV (%)	78 (3)	35 (2.4)	43 (3.6)	0.082
EBV (%)	113 (4.3)	54 (3.7)	59 (4.9)	0.141

Mycoplasma pneumoniae, MP; macrolide-resistant MP, MRMP; Influenza A, IFA; Influenza B, IFB; Chlamydia pneumoniae, CP; Legionella pneumophila, LP; respiratory syncytial virus, RSV; Coxsackie virus, COX; Adenovirus, ADV; Parainfluenza virus, PIV; Epstein–Barr virus, EBV.

**Table 2:** The demographic characteristics and pathogen test results of severely hospitalized children before (2019) and after (2023) the pandemic.

Characteristics	Total (*n* = 361)	2019 (*n* = 54)	2023 (*n* = 307)	*p* Value
Male (%)	180 (49.9)	27 (50)	153 (49.8)	0.982
Age (y)	7.2 (4.9, 9.4)	4.6 (3.1, 7.2)	7.5 (5.8, 9.6)	<0.001
<1 y (%)	15 (4.2)	9 (16.7)	6 (2)	<0.001
1 y–3 y (%)	19 (5.3)	4 (7.4)	15 (4.9)	0.664
3 y–6 y (%)	82 (22.7)	23 (42.6)	59 (19.2)	<0.001
≥6 y (%)	245 (67.9)	18 (33.3)	227 (73.9)	<0.001
Length of stay (d)	7 (6, 9)	9 (7, 12)	7 (6, 9)	0.001
Positive rate (%)	306 (84.8)	35 (64.8)	271 (88.3)	<0.001
Mixed infections (%)	82 (22.7)	8 (14.8)	74 (24.1)	0.133
MP (%)	287 (79.5)	29 (53.7)	258 (84)	<0.001
MRMP (%)	119 (33)	2 (3.7)	117 (38.1)	<0.001
IFA (%)	21 (5.8)	2 (3.7)	19 (6.2)	0.686
IFB (%)	5 (1.4)	4 (7.4)	1 (0.3)	0.001
CP (%)	2 (0.6)	0 (0)	2 (0.7)	1.000
LP (%)	27 (7.5)	0 (0)	27 (8.8)	0.021
RSV (%)	8 (2.2)	0 (0)	8 (2.6)	0.485
COX (%)	3 (0.8)	1 (1.9)	2 (0.7)	0.934
ADV (%)	11 (3)	1 (1.9)	10 (3.3)	0.901
PIV (%)	18 (5)	4 (7.4)	14 (4.6)	0.584
EBV (%)	20 (5.5)	3 (5.6)	17 (5.5)	1.000

Mycoplasma pneumoniae, MP; macrolide-resistant MP, MRMP; Influenza A, IFA; Influenza B, IFB; Chlamydia pneumoniae, CP; Legionella pneumophila, LP; respiratory syncytial virus, RSV; Coxsackie virus, COX; Adenovirus, ADV; Parainfluenza virus, PIV; Epstein–Barr virus, EBV.

**Table 3:** The characteristics of pediatric inpatients with macrolide-resistant Mycoplasma pneumoniae.

Characteristics	MRMP (*n* = 195)	Non-Severe Cases (*n* = 76)	Severe Cases (*n* = 119)	*p* Value
Male (%)	99 (50.8)	40 (52.6)	59 (49.6)	0.678
Age (y)	8.7 (6.9, 10)	8.7 (6.5, 10)	8.7 (7, 10.1)	0.589
<1 y (%)	0 (0)	0 (0)	0 (0)	/
1 y–3 y (%)	0 (0)	0 (0)	0 (0)	/
3 y–6 y (%)	21 (10.8)	11 (14.5)	10 (8.4)	0.182
≥6 y (%)	174 (89.2)	65 (85.5)	109 (91.6)	0.182
Length of stay (d)	6 (5, 8)	5 (4, 6)	7 (5, 9)	<0.001
IFA (%)	13 (6.7)	6 (7.9)	7 (5.9)	0.583
IFB (%)	1 (0.5)	1 (1.3)	0 (0)	0.390
CP (%)	1 (0.5)	1 (1.3)	0 (0)	0.390
LP (%)	19 (9.7)	8 (10.5)	11 (9.2)	0.768
RSV (%)	1 (0.5)	0 (0)	1 (0.8)	1.000
COX (%)	1 (0.5)	0 (0)	1 (0.8)	1.000
ADV (%)	7 (3.6)	4 (5.3)	3 (2.5)	0.542
PIV (%)	8 (4.1)	3 (3.9)	5 (4.2)	1.000
EBV (%)	17 (8.7)	7 (9.2)	10 (8.4)	0.846

Macrolide-resistant Mycoplasma pneumoniae, MRMP; Influenza A, IFA; Influenza B, IFB; Chlamydia pneumoniae, CP; Legionella pneumophila, LP; respiratory syncytial virus, RSV; Coxsackie virus, COX; Adenovirus, ADV; Parainfluenza virus, PIV; Epstein–Barr virus, EBV.

**Table 4:** The percentages of severe cases of each pathogen before (2019) and after (2023) the pandemic.

Pathogens	Total Number of Positive Cases	Percentage of Severe Cases in 2019 (%)	Percentage of Severe Cases in 2023 (%)	*p* Value
MP	1194	5.1 (29/573)	41.5 (258/621)	<0.001
IFA	166	6.1 (2/33)	14.3 (19/133)	0.327
IFB	96	4.9 (4/82)	7.1 (1/14)	0.554
CP	10	0 (0/3)	28.6 (2/7)	1.000
LP	86	/ (0/0)	31.4 (27/86)	/
RSV	42	0 (0/12)	26.6 (8/30)	0.080
COX	34	3.4 (1/29)	40 (2/5)	0.050
ADV	45	7.1 (1/14)	32.3 (10/31)	0.150
PIV	78	11.4 (4/35)	32.6 (14/43)	0.053
EBV	113	5.6 (3/54)	28.8 (17/59)	0.003

Mycoplasma pneumoniae, MP; Influenza A, IFA; Influenza B, IFB; Chlamydia pneumoniae, CP; Legionella pneumophila, LP; respiratory syncytial virus, RSV; Coxsackie virus, COX; Adenovirus, ADV; Parainfluenza virus, PIV; Epstein–Barr virus, EBV.

## Data Availability

The data that support the findings of this study are available upon request from the corresponding author.
